# Genome-Wide Identification of the *DOG1* Gene Family in Pepper (*Capsicum annuum*) and Its Expression Profiles During Seed Germination

**DOI:** 10.3390/plants14131913

**Published:** 2025-06-22

**Authors:** Zhichao Zhao, Jingbo Sun, Feng Zhang, Chunjuan Dong

**Affiliations:** State Key Laboratory of Vegetable Biobreeding, Key Laboratory of Biology and Genetic Improvement of Horticultural Crops (Vegetables), Ministry of Agriculture and Rural Affairs, Institute of Vegetables and Flowers, Chinese Academy of Agricultural Sciences, Beijing 100081, China; 82101235103@caas.cn (Z.Z.); 82101225077@caas.cn (J.S.)

**Keywords:** bioinformatics, pepper (*Capsicum annuum*), *DOG1*, expression profiling, seed germination

## Abstract

The *DOG1* (*Delay of Germination1*) family plays key regulatory roles in seed dormancy and germination. However, a genome-wide analysis of *DOG1* genes has not been performed for pepper (*Capsicum annuum*), one of the agriculturally important species, and no studies have been conducted to characterize their expression profiles. Based on *C. annuum* genome information, the identification and expression analysis of *CaDOG1* gene family members through bioinformatics approaches can provide a theoretical foundation for subsequent studies on the biological functions of *CaDOG1s* and the improvement of seed traits in *C. annuum* breeding. In this study, a total of 13 *CaDOG1* genes were identified in the *C. annuum* genome. Phylogenetic analysis showed that these CaDOG1s, along with DOG1s from thale cress (*Arabidopsis thaliana*), rice (*Oryza sativa*), and maize (*Zea mays*), were classified into four subgroups. All *CaDOG1* genes were unevenly distributed on six *C. annuum* chromosomes, and they had relatively conserved exon–intron patterns, most with zero to one intron. According to the chromosomal distribution patterns and synteny analysis of the *CaDOG1* genes, the *CaDOG1* family expanded mainly through replication, which occurred predominantly after the divergence of dicotyledons and monocotyledons. Conserved motif analysis indicated that all encoded proteins contained Motif 2 and Motif 6, except for CaDOG1-3. Expression profile analysis using transcriptome data revealed that *CaDOG1* genes were differentially expressed across various tissues and developmental stages, with notable involvement in flowers and seeds. Quantitative real-time PCR also revealed that all *CaDOG1* genes were downregulated during seed germination, indicating that *CaDOG1s* may play negative roles in seed germination. Moreover, upon abscisic acid treatment, six *CaDOG1* genes exhibited upregulation, while in response to ethylene, four *CaDOG1* genes exhibited downregulation. Taken together, these findings provide an extensive description of the *C. annuum DOG1* gene family and might facilitate further studies for elucidating their functions in seed germination.

## 1. Introduction

Seed dormancy and germination are fundamental processes in plant biology that have been extensively studied. Dormancy prevents germination under unfavorable conditions, while germination involves metabolic reactivation and embryonic growth [1,2]. Research into dormancy and germination has identified multiple factors contributing to these processes, including inhibitory compounds in the seed coat or embryo, the requirement for cold stratification, environmental cues such as light and temperature, and genetic regulation [3,4,5]. Inhibitory compounds like abscisic acid (ABA) maintain dormancy by regulating gene expression [6,7,8]. Cold stratification, crucial for seeds in temperate regions, can break dormancy and promote germination by altering gene expression [9,10]. Environmental cues such as light and temperature also play key roles. For instance, red light-activated phytochrome B promotes germination by modulating the balance of ABA and GA, and temperature affects germination rate and dormancy release [11,12]. Genetic regulation is essential for seed dormancy and germination. In thale cress (*Arabidopsis thaliana*), the progressive chromatin silencing of ABA biosynthesis genes, regulated by factors like RZ-1 and PRC2, is vital for germination. Mutations in these regulators can cause abnormal phenotypes [7].

*Delay of Germination 1* (*DOG1*) plays a crucial role in regulating seed dormancy and germination in plants. Research into *DOG1* has revealed its importance in controlling the duration and depth of dormancy, as well as the responsiveness of seeds to environmental cues [13]. Variations in the *DOG1* gene sequence have been associated with differences in dormancy levels among different plant species and varieties [14]. Furthermore, studies have shown that *DOG1* expression can be regulated by environmental factors such as temperature and light, as well as by plant hormones like ABA and ethylene (ET) [13]. In *A. thaliana*, expression levels of *AtDOG1* are positively correlated with the degree of seed dormancy and are significantly promoted by low temperature during seed maturation [15,16]. *AtDOG1* enhances ABA signaling by binding to and inhibiting ABA-hypersensitive germination proteins (AHG1/AHG3) and inhibiting specific protein phosphatase 2C (PP2C), thereby inhibiting seed germination [17]. In addition, there is a positive feedback regulation between *AtDOG1* and the ABA-mediated sugar signaling pathway, where glucose induces *AtDOG1* expression, which in turn enhances the expression of *Abscisic Acid Insensitive 4* (*ABI4*), a key gene in the ABA signaling pathway [18]. Furthermore, *DOG1* also interacts with the ET signaling pathway to regulate seed dormancy and germination, and the ETR1/RDO3-ERF12-TPL-DOG1 module may be involved in this process [19]. *DOG1* has also been suggested to act as an environmental sensor of temperature and light, and to regulate the time window of dormancy and germination of seeds. For example, low temperature stress significantly promotes *AtDOG1* expression, leading to higher dormancy levels in *A. thaliana* seeds [20], and high temperature stress also upregulates *AtDOG1* transcript levels and inhibits seed germination by decreasing SUMOylation levels of ALFIN1-like 6 (AL6), resulting in downregulation of AL6 protein levels [21]. Additionally, expression of *AtDOG1* antisense transcripts has shown higher drought sensitivity [22], indicating the potential roles of *DOG1* in environmental adaptation.

In all studied plants, DOG1s are encoded by a multi-gene family. For example, In *A. thaliana*, there are six *DOG1* genes, including *AtDOG1* and five *DOG1-LIKE* genes (*AtDOGL1*, *AtDOGL2*, *AtDOGL3*, *AtDOGL4*, and *AtDOGL5*) [14,21]. A total of 20 *GmDOG1L* genes were identified in soybean (*Glycine max*), 24 *TaDOG1L* genes in common wheat (*Triticum aestivum*), and 3 *OsDOG1L* genes in rice (*Oryza sativa*). Furthermore, *DOGL6* has been found in various angiosperms, such as black cottonwood (*Populus trichocarpa*), maize (*Zea mays*), *T. aestivum*, pineapple (*Ananas comosus*), and so on [14,23,24]. In Arabidopsis, the *dogl4* mutant exhibited slow seed germination and hypersensitivity to ABA, which correlates with the absence of expression of its maternal imprinted gene *AtDOGL4* in the endosperm. Overexpression of *AtDOGL4* reduced seed ABA sensitivity [25,26]. Although no significant germination phenotype was observed in single mutants of *dogl1*, *dogl2*, or *dogl3* [27], overexpression of *AtDOGL3* enhanced seed ABA sensitivity similarly to *AtDOG1* [28]. In the mangrove family, the loss or variation of *DOG1* and *DOGL4* genes is closely associated with the phenomenon of vivipary [29]. In moss (*Physcomitrium patens*), the DOG1-like protein PpDOG1-L1 was found to interact with PpDELLAa, PpDELLAb, and inhibit spore germination by repressing the expression of spore germination-promoting genes [30]. Beyond seed germination, *DOG1* can also regulate seed dormancy and flowering time in lettuce (*Lactuca sativa*) and *A. thaliana* through effects on the levels of miR156 and miR172 [31].

Pepper (*Capsicum annuum*) is cultivated on approximately 3.9 million hectares worldwide and serves as a vital global crop, valued for its multiple roles as a vegetable, spice, phytopharmaceutical, and pigment source [32,33]. The availability of multiple high-quality *C. annuum* genomes (e.g., ‘Zunla-1_v3.0’ and ‘CM334’) has significantly accelerated gene discovery and functional characterization [34,35,36]. In this study, we identified and analyzed the *DOG1* gene family in *C. annuum* (*CaDOG1s*), focusing on their potential roles during seed germination. Through genome-wide identification, we characterized the gene structure, physicochemical properties, phylogenetic relationships, evolutionary relationships, and promoter elements of *CaDOG1* genes. In addition, the in silico and experimental expression patterns of the *CaDOG1* genes were analyzed, providing valuable information for further exploration of the functions of this gene family.

## 2. Results

### 2.1. Identification and Physicochemical Property of CaDOG1 Genes

**Figure 1:**
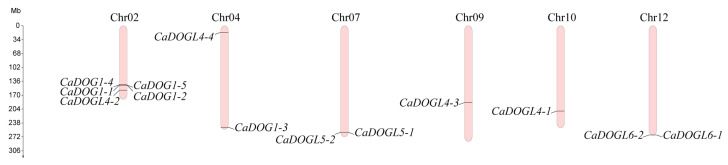
Chromosome localization of *CaDOG1* genes. The chromosome numbers are indicated above each vertical bar, and the scale of the chromosomes is in megabases (Mb).

By using eight *A. thaliana* DOG1 proteins (AtDOG1s) as query sequences for BLASTP, 26 putative *CaDOG1* genes were identified from the *C. annuum* genome database [14]. However, 13 genes encoded the proteins containing a bZIP domain, which belonged to the TGA proteins (Table S1) [26,37]. Therefore, 13 *CaDOG1* genes were identified in *C. annuum*, and according to their homologs in *A. thaliana*, these 13 *CaDOG1* genes were named *CaDOG1-1* to *CaDOGL6-1* (Table 1). These *CaDOG1* genes are distributed across six chromosomes (Figure 1), showing an uneven distribution pattern. Five *CaDOG1* genes are located on Chr02. On Chr04, Chr07, and Chr12, there are two *CaDOG1* genes on each chromosome. Chr09 and Chr10 each contain a single *CaDOG1* gene. Three tandemly arranged gene pairs were identified: *CaDOG1-1*/*CaDOG1-2*, *CaDOGL5-1*/*CaDOGL5-2*, and *CaDOGL6-1*/*CaDOGL6-2*. 

CaDOG1 proteins ranged in size from 116 to 260 amino acids (AA), with a maximum molecular weight (MW) of 30.24 kDa (CaDOGL6-1 and CaDOGL6-2) and a minimum of 13.31 kDa (CaDOG1-4). The isoelectric point (p*I*) of these proteins ranged from 4.55 to 6.15, and the grand average of hydropathicity (GRAVY) was −0.810 to −0.352, indicating the hydrophilicity of these CaDOG1 proteins (Table 1). Subcellular localization predictions indicated that CaDOG1-1, CaDOG1-2, CaDOGL4-1, CaDOGL4-4, and CaDOGL5-1 were localized in the cytoplasm; CaDOG1-4, CaDOG1-5, CaDOGL6-1, and CaDOGL6-2 in the nucleus; CaDOGL4-3, and CaDOGL5-2 in mitochondria; CaDOGL4-2 in chloroplasts; and CaDOG1-3 exhibited dual cytoplasmic–nuclear distribution.

### 2.2. Phylogenetic Analysis of CaDOG1 Proteins

**Figure 2:**
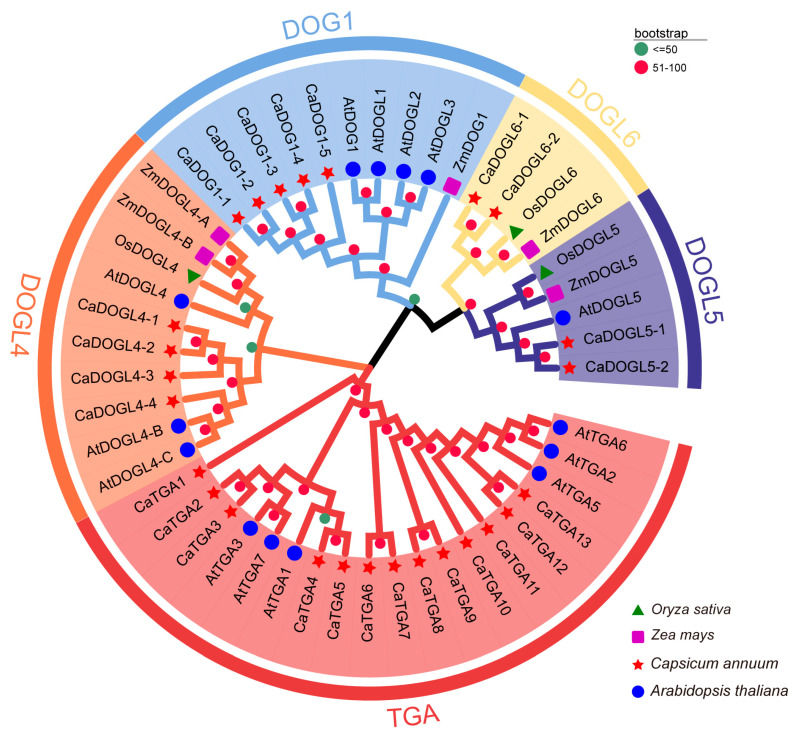
Phylogenetic analysis of DOG1 proteins from different plant species. The DOG1 proteins from thale cress (*Arabidopsis thaliana*, AtDOG1s), pepper (*Capsicum annuum*, CaDOG1s), rice (*Oryza sativa*, OsDOG1s), and maize (*Zea mays*, ZmDOG1s), were aligned using ClustalX 2.0, and the phylogenetic tree was constructed using the Neighbor-Joining (NJ) method with bootstrap values (>50) of 1000 replications.

To evaluate the phylogenetic relationships of the CaDOG1 proteins with other plant DOG1s (Table S1), an NJ phylogenetic tree was constructed based on the protein sequence alignments of a set of DOG1 family members from other plant species, including *C. annuum*, *A. thaliana*, and two monocot plants (*O. sativa* and *Z. mays*). As shown in Figure 2, the resulting tree divided these DOG1 proteins into four groups (DOG1, DOGL4, DOGL5, and DOGL6), and the TAGs were separated into a distinct group.

### 2.3. Intron/Exon Organization and Conserved Motif Analysis of CaDOG1 Genes

**Figure 3:**
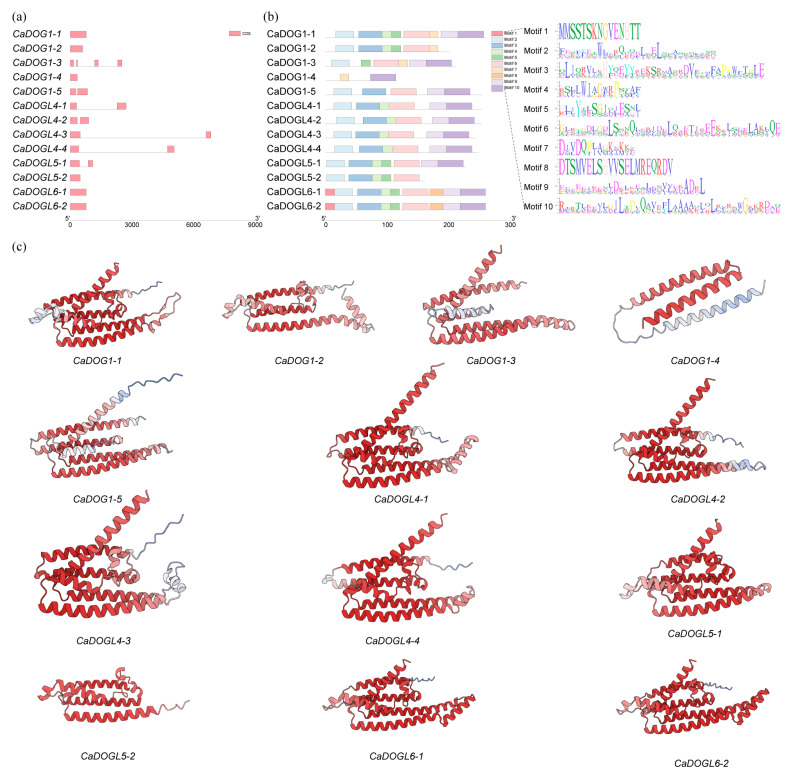
Gene structure, motif distribution, and protein structures of CaDOG1s. (**a**) Exon–intron organizations of *CaDOG1* genes. (**b**) Motif distribution of CaDOG1 proteins. (**c**) Predicted 3D structures of CaDOG1 proteins. The protein structure was predicted by AlphaFold3. The darker the color, the more reliable the prediction result.

Gene structure analysis indicated that the number of exons in these genes is relatively stable (Figure 3a). The majority possess one or two exons, and only *CaDOG1-3* contains 4 exons. Genes with a single exon have comparable exon lengths, whereas genes with two exons have similar exon lengths but significant differences in intron lengths. Among them, *CaDOGL4-1*, *CaDOGL4-3*, and *CaDOGL4-4* have introns that are much longer than their exons, while *CaDOG1-5*, *CaDOGL4-2*, and *CaDOGL5-1* have considerably shorter intron lengths.

Motif analysis showed that most CaDOG1 proteins contain five to nine motifs, with only CaDOG1-4 having two motifs, (Figure 3b). Motif 7 is exclusive to the DOG1 group. The proteins in the DOGL6 group share the same motif composition, with Motif 1 and Motif 8 being specific to the DOGL6 group. Also, the proteins within the DOGL4 group contain the same motifs, but lack Motif 5, which is present in all of the other groups.

The predicted three-dimensional (3D) structures of CaDOG1 proteins are shown in Figure 3c. All CaDOG1 proteins, except for CaDOG1-4, exhibit a predominantly α-helical secondary structure with limited random coil regions, suggesting high structural stability. For CaDOG1-4, the structure reliability (pTM) was lower than 0.5. A central structural motif composed of α-helices and random coils corresponds to the conserved DOG1 domain.

### 2.4. Identity and Collinearity Analysis of CaDOG1 Family Genes

**Figure 4:**
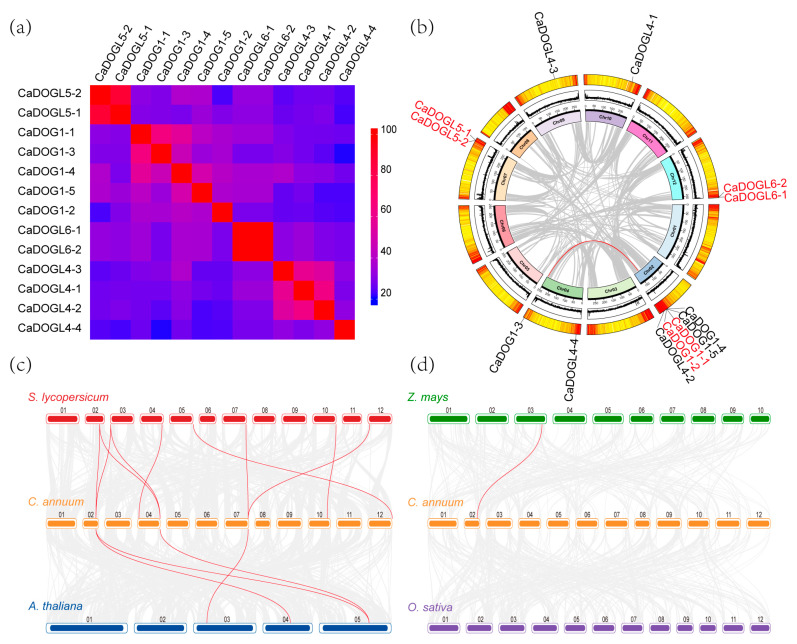
Analysis of similarity and collinearity of *CaDOG1* family genes. (**a**) Protein sequence identity within the CaDOG1 family. (**b**) Collinearity analysis of *CaDOG1* family genes. The outermost circular ring stands for the gene density on the chromosome. The greater the number of red lines, the denser the genes are. The middle circle represents the GC content of the chromosome nucleotide sequence, where higher black lines signify a higher GC content. The innermost ring indicates the chromosome name as well as its length. (**c**) Collinearity analysis of *DOG1* family genes among *C. annuum*, *S. lycopersicum*, and *A. thaliana*. (**d**) Collinearity analysis of *DOG1* family genes among *C. annuum*, *Z. mays*, and *O. sativa*. The red lines highlight the syntenic *DOG1* gene pairs.

Gene duplication plays a crucial role in species evolution, with segmental and tandem duplications being considered the two main mechanisms for expanding gene families [38]. The evolutionary relationship of the *CaDOG1* gene family was analyzed based on sequence identity and collinearity analysis. As shown in Figure 4a, CaDOG1 proteins exhibit 20.8–100% identity. Notably, the protein sequence identity between CaDOGL5-1 and CaDOGL5-2 is as high as 88.1%, and the amino acid sequences of CaDOGL6-1 and CaDOGL6-2 are identical, while the lowest identity was observed between CaDOG1-2 and CaDOGL5-1. Collinearity analysis revealed that *CaDOG1-3* and *CaDOG1-5* were collinear in the genome (Figure 4b), suggesting a shared ancestral origin. The protein pairs CaDOGL5-1/CaDOGL5-2, CaDOGL6-1/CaDOGL6-2, and CaDOG1-1/CaDOG1-2 show 66.4–100% sequence identity and are organized in tandem arrays, suggesting they originated through tandem duplication events. The identity between CaDOG1-1 and CaDOG1-2 is 66.4%, which might be attributed to the fact that, compared to the other two pairs of tandem repeat genes, *CaDOG1-1* and *CaDOG1-2* underwent gene replication at a relatively earlier stage. The evidence presented above indicates that gene replication has occurred in the *CaDOG1* gene family, suggesting that the *CaDOG1* genes may have expanded the family through replication during evolution.

This study further investigated collinearity between *CaDOG1* genes in *C. annuum* and other species. We constructed a multi-species collinearity diagram of *DOG1* family genes among tomato (*Solanum lycopersicum*), *A. thaliana*, *C. annuum*, *Z. mays*, and *O. sativa* (Figure 4c, d). The results showed that, when compared with *S. lycopersicum*, *A. thaliana*, and *Z. mays*, *CaDOG1* genes had 9, 4, and 1 homologous gene pairs with the *DOG1* genes in those species, respectively. However, no homologous gene pairs were found with *O. sativa*. Notably, *CaDOG1-5* harbors homologous genes with *S. lycopersicum*, *A. thaliana*, and *Z. mays*, suggesting that *CaDOG1-5* exhibits greater conservation among plants compared to other *CaDOG1* genes. In addition, it was also found that the number of homologous gene pairs between *C. annuum* and two dicotyledonous plants (*S. lycopersicum* and *A. thaliana*) is significantly higher than that between two monocotyledonous plants (*Z. mays* and *O. sativa*), indicating that the *CaDOG1* gene family likely originated from the divergence between monocotyledonous and dicotyledonous plants and exhibits better conservation with dicotyledonous plants. Further analysis of the collinearity between *C. annuum* and two dicotyledonous plants reveals that the number of homologous gene pairs between *C. annuum* and *S. lycopersicum* is significantly higher than that with *A. thaliana*, further validating their genetic relationship. Specifically, *C. annuum* and *S. lycopersicum*, both members of the Solanaceae family, exhibit higher evolutionary affinity compared to the other three species.

### 2.5. cis-Element Analyses of CaDOG1 Gene Family

**Figure 5:**
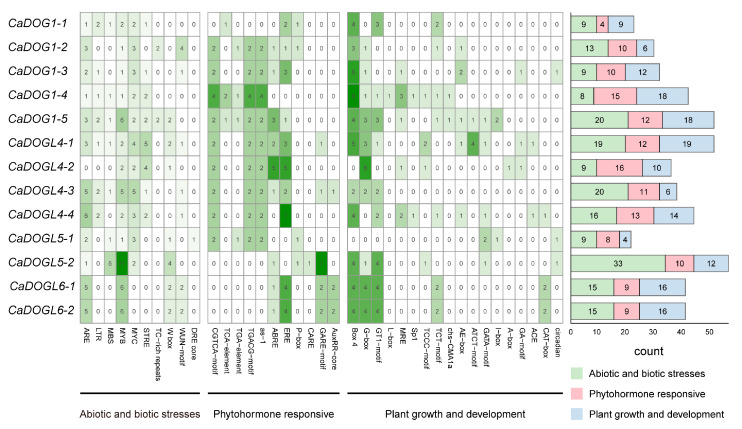
*cis*-element analysis of *CaDOG1* promoters. Blocks of different colors and quantities represent the number of different promoter elements contained within the upstream region of the *CaDOG1* genes at 2000 bp. Darker colors indicate a higher number of promoter elements. The different-colored histogram represents the sum of the *cis*-acting elements in each category.

*cis*-elements are recognized as one of the crucial factors in regulating gene expression [39]. To investigate the *cis*-regulatory structures of *CaDOG1* promoters, 2000 bp upstream of the translational start of each *CaDOG1* gene was analyzed using PlantCARE. The identified *cis*-elements were then classified and statistically analyzed into three categories: abiotic and biotic stress response, plant hormone response, and plant growth and development (Figure 5) [40].

The category of abiotic- and biotic-stress-responsive *cis*-elements included elements related to various stresses, such as drought (MBS and DRE), high temperature (STRE), low temperature (LTR), salt (DRE), wounds (WUN and TC-rich), and anaerobic stress (ARE), as well as some common stress-responsive elements (MYB and MYC). MYB, MYC, and ARE can be found in almost all genes and account for 29%, 16%, and 19% of the *cis*-elements in this category, respectively.

The category of phytohormone-responsive elements included CGTCA and TGACG for jasmonic acid (JA); TCA and as-1 for salicylic acid (SA); CARE, the GARE motif, and P-box for gibberellin (GA); ABRE for ABA; TGA and AuxRR core for auxin (IAA); and ERE for ET. The presence of these phytohormone-responsive elements indicated that the expression of these genes may be regulated by multiple phytohormones, including JA, SA, GA, ABA, IAA, and ET, suggesting their potential roles in diverse hormonal signaling pathways.

In the plant growth and development category, 18 *cis*-elements were identified, and 15 elements were related to light response. Among them, Box 4 was the most abundant (30%, and in the promoter regions of 11 *CaDOG1* genes). Also, the RY-element for seed-specific expression, the circadian element for circadian rhythm regulation, and the CAT-box for meristematic expression were identified.

### 2.6. Tissue-Specific Expression Patterns of CaDOG1 Genes in C. annuum

**Figure 6:**
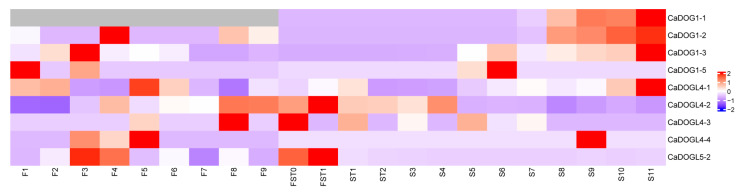
Expression patterns of *CaDOG1* genes in different tissues of *C. annuum*. The expression levels of the *CaDOG1* gene family were displayed based on RPKM-standardized values (Table S2). F1 to F9 represent flowers with lengths of 2.5, 3.5, 5.0, 7.0, 8.0, 10.0, 12.0, 14.5, and 17.0 mm. FST0 to S11 correspond to fruits collected at 3, 7, 10, 15, 20, 25, 30, 35, 40, 45, 50, 55, and 60 days post-anthesis, from which placental tissues and seeds were dissected. FST denotes the flower, seed, and placenta complex; ST denotes the seed and placenta complex; and S denotes the seed.

Based on publicly available transcriptome data from various *C. annuum* tissues matched with Zunla-1_V3.0, the expression patterns of nine *CaDOG1* genes were analyzed in different tissues, including flowers, placenta, and seed at various developmental stages (Table S2) [41]. Each *CaDOG1* gene exhibited significant spatiotemporal expression specificity (Figure 6). The expression levels of various genes exhibited distinct dynamic changes during developmental progression. In flower tissues, the expression of *CaDOG1-2*, *CaDOG1-3*, *CaDOGL4-4*, and *CaDOGL5-2* increased at the mid-developmental stage and subsequently declined. In contrast, *CaDOG1-5* showed reduced expression in the early developmental stage and then maintained a stable level thereafter. The expression of *CaDOGL4-1* gradually decreased in the early stage, increased during mid-development, and then declined again. Meanwhile, *CaDOGL4-2* and *CaDOGL4-3* displayed an initial increase followed by a decrease and then another rise in expression from the mid- to late-developmental stages. During the early stages of fruit development (FST0–ST2), the expression levels of *CaDOGL4-1* and *CaDOGL4-2* initially increased and then decreased. *CaDOGL4-3* exhibited an initial downregulation, followed by upregulation and subsequent downregulation. In contrast, *CaDOGL5-2* showed a continuous decrease in expression throughout fruit development. The expression levels of the remaining genes remained unchanged during this period. In seed tissues, the expression levels of *CaDOG1-1*, *CaDOG1-2*, *CaDOG1-3*, and *CaDOGL4-1* were consistently upregulated during seed development, whereas *CaDOGL4-2* and *CaDOGL4-3* were downregulated. The remaining genes were either not expressed or only detectable at specific developmental stages. The differential expression profiles imply that *CaDOG1* genes function critically in seed maturation, dormancy regulation, and flower development.

### 2.7. Expression Patterns of CaDOG1 Genes During Seed Germination

**Figure 7:**
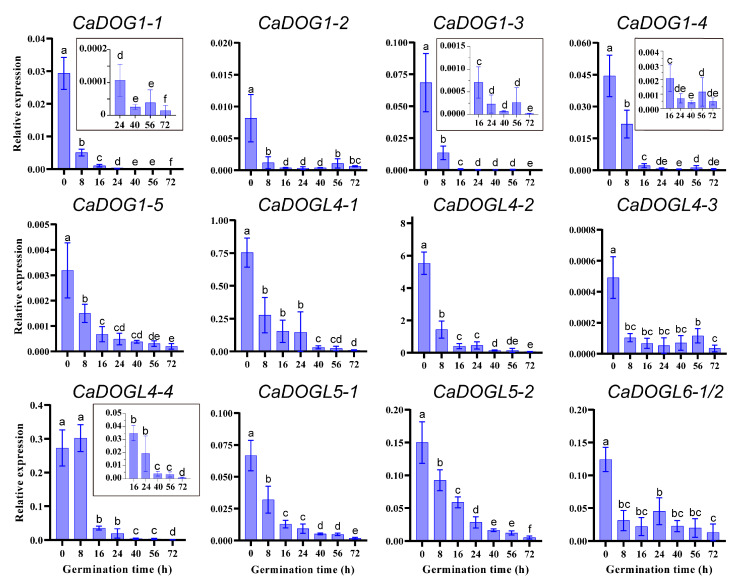
Expression profiles of *CaDOG1* genes during *C. annuum* seed germination. The expression levels of each *CaDOG1* gene were quantified by qRT-PCR and normalized to *CaActin-7*. All experiments were conducted in triplicate with at least three independent biological replicates. Error bars indicate the standard error of the mean. The different lowercase letters indicate significant differences (*p* < 0.05), as determined using one-way ANOVA followed by the Waller–Duncan test. The sequences of *CaDOGL6-1* and *CaDOGL6-2* are identical; therefore, they are presented collectively in this figure.

Previous studies have demonstrated that the *DOG1* can delay seed germination [13]. To characterize the functions of CaDOG1 members in *C. annuum* seed germination, quantitative real-time polymerase chain reaction (qRT-PCR) analysis was carried out on the *CaDOG1* genes in *C. annuum* seeds at different germination time points (0, 8, 16, 24, 40, 56, and 72 h). As shown in Figure 7, 13 *CaDOG1* genes showed similar expression patterns. Specifically, the expression levels of most *CaDOG1* genes exhibited a substantial decline by 8 h after germination, with the exception of *CaDOGL4-4*, whose expression did not decline significantly until 16 h. Notably, in seeds, *CaDOGL4-2* showed the highest expression levels, consistent with the previous transcriptomic data (Table S2). These findings suggest that *CaDOG1* genes may exert negative effects on seed germination in *C. annuum*.

### 2.8. Hormone-Responsive Expression Patterns of CaDOG1 Genes During Seed Germination

**Figure 8:**
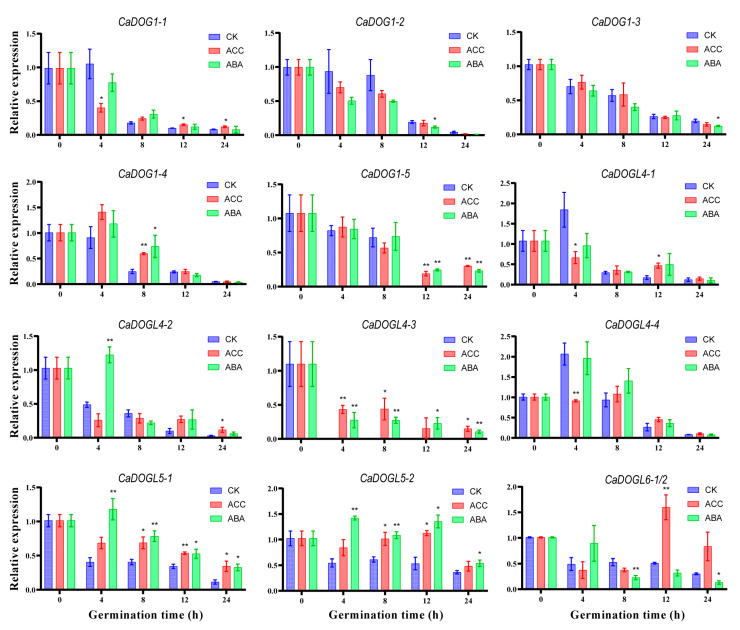
Expression profiles of *CaDOG1* genes in response to ACC and ABA during pepper seed germination. The expression levels of each *CaDOG1* gene were quantified by qRT-PCR. The expression level for each gene in the CK plants at 0 h was normalized to 1.0. All experiments were conducted in triplicate with at least three independent biological replicates. Error bars indicate the standard error of the mean. A *t*-test was used to determine statistically significant differences in the expression levels under different conditions compared to CK (* *p* < 0.05, ** *p* < 0.01). The sequences of *CaDOGL6-1* and *CaDOGL6-2* are identical and therefore are presented collectively in this figure.

*DOG1* expression was reported to be associated with ET and ABA signaling pathways in *A. thaliana*, hedge mustard (*Sisymbrium officinale*)*,* and *Lepidium* [13,19,42]. In addition, some ERE and ABRE elements were predicted in the *CaDOG1* gene promoters (Figure 5). Therefore, we conducted qRT-PCR to analyze the expression of *CaDOG1* genes in the *C. annuum* seeds treated with 1-aminocyclopropane-1-carboxylic acid (ACC, an ethylene precursor) and ABA, as illustrated in Figure 8. The concentrations were determined by referring to previous studies and preliminary experimental results (Figure S1).

Upon ACC treatment, *CaDOG1-1*, *CaDOGL4-1*, and *CaDOGL4-4* were downregulated at 4 h of treatment, while *CaDOGL4-3* was upregulated. After 8 h, the majority of genes exhibited upregulation at various time points. Notably, *CaDOGL4-3, CaDOGL5-1,* and *CaDOGL5-2* remained upregulated at all time points after 8 h.

ABA repressed the expression of *CaDOG1-2, CaDOG1-3,* and *CaDOGL6-1/2* during seed germination. In contrast, *CaDOG1-4*, *CaDOG1-5, CaDOGL4-2*, *CaDOGL4-3, CaDOGL5-1*, and *CaDOGL5-2* exhibited upregulation after ABA treatment.

The antagonistic and synergistic responses of *CaDOG1* genes to ACC and ABA demonstrate their specialized roles in germination regulation.

## 3. Discussion

DOG1 proteins play a pivotal role in regulating seed dormancy and germination across various plant species [43]. This role is often intertwined with other key regulators, such as ABI3/4/5, ETR1, and ERF12, which jointly modulate hormonal pathways to fine-tune dormancy depth [19,44,45,46]. In this study, we identified and characterized the *CaDOG1* gene family in *C. annuum*, providing some comprehensive insights into its structural features, evolutionary relationships, and expression patterns during seed germination and other developmental stages.

Phylogenetic analysis revealed that the *CaDOG1* genes can be classified into four distinct groups, consistent with other plant species such as *A. thaliana* and *O. sativa a*. This conservation suggests that the *DOG1* gene family has maintained its core regulatory functions throughout plant evolution [14,42,47,48,49]. The presence of tandem and segmental duplications within the *CaDOG1* gene family suggests that gene duplication has been a key mechanism driving the expansion and diversification of this gene family during evolution. Notably, the high sequence identity between *CaDOGL5-1* and *CaDOGL5-2*, as well as the identical sequences of *CaDOGL6-1* and *CaDOGL6-2*, indicates possible functional redundancy or subfunctionalization among these genes.

The uneven distribution of *CaDOG1* genes across *C. annuum* chromosomes further supports the idea that these genes have evolved through species-specific adaptation [14]. For example, the high concentration of *CaDOG1* genes on chromosome 2 suggests that this region may be a hotspot for gene duplication and functional innovation [38]. The presence of conserved motifs, such as Motif 7 in the DOG1 group and Motif 1 and Motif 8 in the DOGL6 group, highlights the potential functional specialization of these subgroups. These motifs may serve as binding sites for transcription factors or other regulatory proteins, enabling fine-tuned control of seed dormancy and germination in response to environmental cues [50,51,52]. Additionally, the predicted 3D structures of CaDOG1 proteins indicate a high degree of structural conservation, further supporting their functional relevance in seed dormancy and germination.

The presence of stress-responsive and hormone-related *cis*-elements in the promoter regions of *CaDOG1* genes supports their role in integrating environmental and hormonal signals to regulate seed germination. For example, the abundance of ABRE and ERE suggests that *CaDOG1* genes are tightly regulated by the antagonistic actions of ABA and ET, which are key hormones controlling seed dormancy and germination [17,19,28,53,54,55,56]. Interestingly, the expression of *CaDOG1* genes was also influenced by temperature and light, as evidenced by the presence of low-temperature-responsive elements (LTR) and light-responsive elements (Box 4) in their promoters. For example, in *A. thaliana*, low-temperature stress during seed maturation has been shown to enhance *DOG1* expression, leading to deeper dormancy and delayed germination [20]. Conversely, warm temperatures and light exposure can reduce *DOG1* expression, promoting germination under favorable conditions [16]. Overexpression of the *T. aestivum DOG1-1* gene (*TaDOG1-1*) in *A. thaliana* has been shown to improve heat tolerance [24]. Under salt stress, DOG1-deficient (*dog1-3*) and overexpressors (*dog1-5*) showed faster and slower germination, respectively [57]. This regulatory paradigm mirrors the interaction between *DOG1* and *PHYTOCHROME INTERACTING FACTORS* (*PIFs*) in light-mediated germination control, where *PIF3-LIKE 5* (*PIL5*)/*PIF1* can directly activate the transcription of *GA-INSENSITIVE* (*GAI*) and *REPRESSOR OF ga1-3* (*RGA*), thereby inhibiting GA signal transduction and suppressing seed germination. Additionally, it indirectly inhibits the biosynthesis of GA (*GA3ox1 and GA3ox2*) through its downstream effectors *SOMNUS* (*SOM*) and *DOF AFFECTING GERMINATION 1* (*DAG1*), while activating the biosynthesis of ABA (*ABA1*, *NCED6*, and *NCED9*) [58]. This suggests that *CaDOG1* genes may act as environmental sensors, modulating seed dormancy and germination in response to seasonal changes and other environmental factors.

The expression analysis of *CaDOG1* genes across different tissues and developmental stages revealed distinct expression patterns, suggesting they play a key role in seed maturation and dormancy balance, as well as in flower development. RT-PCR analysis also revealed that *CaDOG1* expression levels progressively decreased during seed germination, consistent with the *DOG1* genes in *A. thaliana* and *T. aestivum*. This downregulation suggests that *CaDOG1* genes act as evolutionarily conserved negative regulators of seed germination [27,59,60]. Preliminary evidence suggests that this family may coordinate seed germination and dormancy through functional diversification and feedback regulation, highlighting its potential as a target for further investigation in non-model crops. In addition, genes involved in seed germination have been shown to regulate flowering [61]. For example, in *A. thaliana*, mutations of *HISTONE MONOUBIQUITINATION1* and *2* (*HUB1* and *HUB2*) genes inhibited both seed germination and flowering [62,63,64]. Similarly, in *L. sativa*, *LsDOG1* regulates both seed germination and flowering time by modulating miR156 and miR172 [31]. Consistent with these findings, our study revealed that *CaDOG1-2*, *CaDOG1-3*, *CaDOG1-5*, *CaDOGL4-1*, *CaDOGL4-2*, *CaDOGL4-3*, *CaDOGL4-4*, and *CaDOGL5-2* genes display stage-specific expression patterns during flower development. These findings suggest that *CaDOG1* genes may not only be involved in seed dormancy and germination but may also play roles in the development of flowers.

The dynamic expression patterns of *CaDOG1* genes in response to ACC and ABA treatments suggest that CaDOG1s might be involved in the ET and ABA signaling pathways during *C. annuum* seed germination. Upon ACC treatment, four genes (*CaDOG1-1*, *CaDOGL4-1*, *CaDOGL4-2*, and *CaDOGL4-4*) showed significant downregulation at 4 h, consistent with ethylene’s dormancy-breaking role [65]. Subsequently, most *CaDOG1* genes, including these four, exhibited upregulated expression (either transient or sustained) between 8 and 24 h, suggesting that they are potentially associated with post-germination development. This regulation suggests functional diversification within the *CaDOG1* gene family, where distinct members may coordinate stage-specific responses: early-acting genes mediate dormancy termination through ET signaling, and late-induced genes modulate cellular expansion during seedling establishment [19]. ABA treatment resulted in different expression patterns of *CaDOG1* genes in *C. annuum* seeds, reflecting the complex interaction between ABA signaling and germination regulation. The sustained upregulation of *CaDOGL5-1* and *CaDOGL5-2* throughout the germination process is consistent with the function of conserved *DOG1* in enhancing ABA sensitivity, similar to the *AtDOG1* stable signal complex maintaining dormancy in early developmental stages [17]. These genes may enhance ABA-mediated germination inhibition, ensuring that seeds respond appropriately to environmental signals. On the contrary, the downregulation of *CaDOG1-2* and *CaDOGL6-1/2* may be related to the subfunctional differentiation of the *CaDOG1* gene family. These downregulated genes may act as auxiliary genes, reducing excessive ABA signaling to achieve controlled embryonic root extension and growth, and avoiding excessive seed dormancy. Although the function of *CaDOG1s* is currently considered highly similar to *AtDOG1s*, primarily associated with the ABA/ET signaling pathway, recent studies in *S*. *lycopersicum* have revealed that the transcription factor *SlLBD40* delays endosperm weakening by suppressing the expression of cell wall remodeling enzyme genes (*SlEXP6*, *SlXTH23*, and *SlMAN1*), thereby inhibiting seed germination [66]. Similarly, *CaDOG1s* may also influence seed germination by regulating cell wall metabolism, rather than being limited to the ABA/ET signaling pathway. Future studies could explore whether *CaDOG1s* directly target cell wall remodeling enzyme genes to uncover new mechanisms in seed germination regulation.

In conclusion, this study provides a comprehensive characterization of the *CaDOG1* gene family, including genome-wide identification, phylogenetic classification, and expression profiling under hormonal treatments. Although we have successfully identified and preliminarily characterized the *CaDOG1* family, their precise molecular functions and regulatory mechanisms remain unclear. Building on these findings, future research will prioritize functional validation to elucidate the mechanistic roles of *CaDOG1* genes. Key investigations will include selective silencing and overexpression of candidate genes to assess their effects on seed dormancy, germination, and flowering time regulation. Protein–protein interaction networks, particularly with ABA and ET signaling components, will be systematically mapped through yeast two-hybrid (Y2H) and co-immunoprecipitation assays (Co-IP). Phenotypic analyses under various abiotic stresses (heat, salt, and cold) will further clarify their roles in environmental adaptation. By integrating genomic insights with molecular and physiological validation, these investigations will not only reveal the functional diversification of *CaDOG1* genes but also provide molecular targets for improving germination traits in *C. annuum* breeding programs.

## 4. Materials and Methods

### 4.1. Identification of CaDOG1 Genes

Eight AtDOG1 protein sequences were downloaded from the *A. thaliana* genome database (https://www.arabidopsis.org/) and then used as queries to perform a BLASTP search in the PepperBase database (http://www.bioinformaticslab.cn/PepperBase/ (accessed on 6 November 2024)) [35], using an e-value ≤ 1 × 10^−5^ as a cut-off. The conserved domains of the obtained protein sequences were analyzed using InterPro (https://www.ebi.ac.uk/interpro/search/sequence/ (accessed on 10 November 2024)) [37], and genes encoding bZIP domains were excluded.

### 4.2. Gene Structure and Sequence Analysis of CaDOG1 Genes

The gene ID, chromosome position, protein sequence, CDS sequence, and gene annotation files were downloaded from the PepperBase (http://www.bioinformaticslab.cn/PepperBase/ (accessed on 6 November 2024)) [35]. The locations of the *CaDOG1* genes on the chromosome were visualized using MG2C (http://mg2c.iask.in/mg2c_v2.1/index_cn.html (accessed on 27 November 2024)) [67]. Amino acid length (AA), molecular weight (MW), and isoelectric point (p*I*) of CaDOG1 proteins were calculated using the ProtParam program (https://web.expasy.org/compute_pi/ (accessed on 23 November 2024)) [68,69,70]. The overall average hydrophilicity (GRAVY) was measured using the Sequence Manipulation Suite (https://www.detaibio.com/sms2/protein_gravy.html (accessed on 23 November 2024)) [71]. Subcellular localization of CaDOG1 proteins was predicted using WoLF PSORT (https://wolfpsort.hgc.jp/ (accessed on 23 November 2024)) [72]. The conserved motifs of CaDOG1 proteins were predicted using the online tool MEME 5.5.7 (https://meme-suite.org/meme/tools/meme (accessed on 13 November 2024)) [73], with the number of motifs set to 10 and their lengths ranging from 6 to 50 amino acids. The gene structure and conserved sequences were subsequently visualized using TBtools software (version 2.225). Additionally, the three-dimensional (3D) protein structures were predicted using AlphaFold3 (https://alphafoldserver.com/ (accessed on 2 December 2024)) [74] and visualized using the Protein Viewer extension in VScode (version 1.99.3) [75].

### 4.3. cis-Element Analysis of CaDOG1 Promoters

The upstream 2000 bp sequence from the start codon of the *CaDOG1* genes was extracted as the promoter sequence using TBtools [76]. The *cis*-acting elements within the promoter region were analyzed using PlantCARE (http://bioinformatics.psb.ugent.be/webtools/plantcare/html/ (accessed on 7 November 2024)) [77]. Visualization of the analysis results was performed using TBtools.

### 4.4. Phylogenetic Analysis and Collinearity Analysis

The DOG1 protein sequences of *Z. mays* and *O. sativa* were downloaded from the NCBI database (https://www.ncbi.nlm.nih.gov/). These sequences, along with DOG1s from *C. annuum* and *A. thaliana*, were uploaded to MEGA11 software (version 11.0.13). A phylogenetic tree was constructed using the Neighbor-Joining Algorithm method with 1000 bootstrap replicates. The resulting tree was further visualized and refined using Evolview (https://www.evolgenius.info/evolview/ (accessed on 12 November 2024)) [78]. The sequence identity of the CaDOG1 proteins was analyzed using EMBL-EBI (https://www.ebi.ac.uk/jdispatcher (accessed on 2 March 2025)) [79], and the results were visualized as a heatmap using R-4.4.1 (packages: ComplexHeatmap and circlize). Collinearity analysis within *C. annuum* species, as well as between *C. annuum* and *S*. *lycopersicum*, *A. thaliana*, *Z. mays*, and *O. sativa*, was performed and visualized using the One Step MCscanX tool in TBtools.

### 4.5. In Silico Expression Analysis of CaDOG1 Genes

In silico expression profiles of *CaDOG1* genes were analyzed using the Pepperhub database (http://lifenglab.hzau.edu.cn/PepperHub/index.php (accessed on 2 March 2025)) [41], by retrieving the RPKM values from the database. The transcript expressions were searched against databases using the gene IDs, as detailed in Table S2. The heatmap was generated with R 4.4.1 (packages: ComplexHeatmap and circlize).

### 4.6. Plant Materials and qRT-PCR Analysis of Gene Expression

The *C. annuum* variety used in the experiment was “Guofu 801”, developed by Jingyan Yinong (Beijing) Seed Technology Co., Ltd. Plump seeds were selected and surface-sterilized by soaking in a 5% sodium hypochlorite solution (NaClO) for 10 min, followed by rinsing five times with distilled water. A total of 50 seeds were placed in Petri dishes lined with two layers of absorbent paper and two layers of filter paper, and 7 mL of distilled water was added to each dish. The seeds were then germinated in a dark incubator at 26 °C. After 0, 8, 16, 24, 40, 56, and 72 h of imbibition, the seeds were removed, immediately dried with absorbent paper, and flash-frozen in liquid nitrogen. Subsequently, the samples were stored at −80 °C for subsequent gene expression analysis. For ACC and ABA treatments, *C. annuum* seeds were placed on the paper containing ACC (50 mg/L) or ABA (100 mg/L). Then, the germinating seeds were collected at 0, 4, 8, 12, and 24 h of imbibition. The control seeds were simultaneously treated without additional hormones. Each treatment was performed with three biological replicates to ensure the reliability of the experimental results.

RNA was extracted from *C. annuum* seeds using the RNAprep Pure polysaccharide polyphenol plant total RNA extraction kit (DP441, Tiangen Biotech (Beijing) Co., Ltd., Beijing, China). Reverse transcription was performed using TransScript^®^ Uni All-in-One First-Strand cDNA Synthesis SuperMix for qPCR (One-Step gDNA Removal) (AU341-02, TransGen Biotech Co., Ltd., Beijing, China) to obtain the cDNA. Primers were designed using Primer3Plus (https://www.primer3plus.com/ (accessed on 4 December 2024) and subsequently synthesized and purified by Sangon Biotech. The primer sequences are listed in Table S3. Real-time PCR reactions were conducted on a LightCycler^®^ 96 instrument (Roche Diagnostics GmbH, Mannheim, Germany) with the PerfectStart^®^ Green qPCR SuperMix kit (AQ601, TransGen Biotech Co., Ltd.). The PCR program consisted of an initial denaturation at 95 °C for 3 min, followed by 45 cycles of denaturation at 95 °C for 5 s, annealing at 58 °C for 10 s, and extension at 72 °C for 10 s. The *CaACTIN-7* gene (ZLC03G0027560.1) was used as an internal reference, and the relative gene expression levels were calculated using the 2^−∆Ct^ method and 2^−∆∆Ct^ method [80].

### 4.7. Statistical Analysis

Statistical analysis was performed using SPSS (version 27.0). Data were analyzed using one-way analysis of variance (ANOVA), and significant differences between groups were determined using the Waller–Duncan test and *t*-test. Normality and homogeneity of variance were also tested. The significance level was set at *p* < 0.05. Graphs were generated using GraphPad Prism9.

## Figures and Tables

**Table 1:** Characteristics of CaDOG1s.

Name	Gene ID	Chr.	AA	MW (kDa)	p*I*	Gravy	Subcellular Localization
*CaDOG1-1*	ZLC02G0015410	2	260	29.80	5.48	−0.560	cyto
*CaDOG1-2*	ZLC02G0015420	2	202	22.69	5.11	−0.484	cyto
*CaDOG1-3*	ZLC04G0026780	4	210	24.11	5.19	−0.605	cyto_nucl
*CaDOG1-4*	ZLC02G0015240	2	116	13.31	6.06	−0.810	nucl
*CaDOG1-5*	ZLC02G0015390	2	254	28.90	6.00	−0.647	nucl
*CaDOGL4-1*	ZLC10G0015190	10	253	29.08	4.55	−0.516	cyto
*CaDOGL4-2*	ZLC02G0022320	2	253	28.94	4.83	−0.352	chlo
*CaDOGL4-3*	ZLC09G0015000	9	243	28.28	5.83	−0.456	mito
*CaDOGL4-4*	ZLC04G0008420	4	248	28.39	5.30	−0.385	cyto
*CaDOGL5-1*	ZLC07G0022880	7	227	26.41	5.15	−0.425	cyto
*CaDOGL5-2*	ZLC07G0022870	7	161	18.88	6.15	−0.361	mito
*CaDOGL6-1*	ZLC12G0030560	12	260	30.24	5.86	−0.664	nucl
*CaDOGL6-2*	ZLC12G0030550	12	260	30.24	5.86	−0.664	nucl

## Data Availability

All data in this study can be found in the manuscript or the Supplementary Materials.

## Supplementary Materials

The following supporting information can be downloaded at https://www.mdpi.com/article/10.3390/plants14131913/s1, Table S1: Gene IDs of *CaTGAs*, *AtDOG1s*, *OsDOG1s*, and *ZmDOG1s*; Table S2: RPKM standardized values of *CaDOG1* genes; Table S3: The primers used in this study and their corresponding gene IDs; Figure S1: Germination of pepper seeds under dH2O (CK), ACC, and ABA treatments.
