# Supplementary material for: Genome-Wide Identification of the DOG1 Gene Family in Pepper (Capsicum annuum) and Its Expression Profiles During Seed Germination
Source: Plants (Basel). 2025 Jun 22;14(13):1913. doi: 10.3390/plants14131913 (PMC12251688; doi:10.3390/plants14131913)
Supplement: Supplementary file 1 [file plants-14-01913-s001.zip › Figure S1 Germination of pepper seeds under dH2O (CK), ACC and ABA treatments..pdf]

(a)

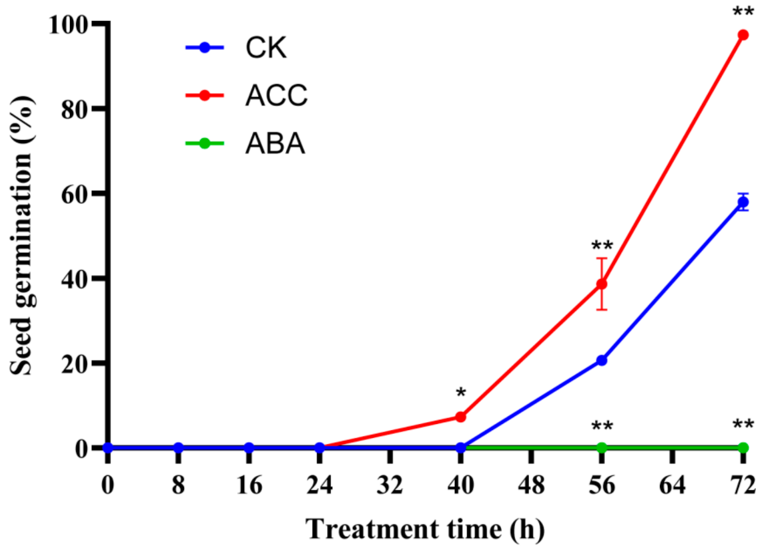

(b)

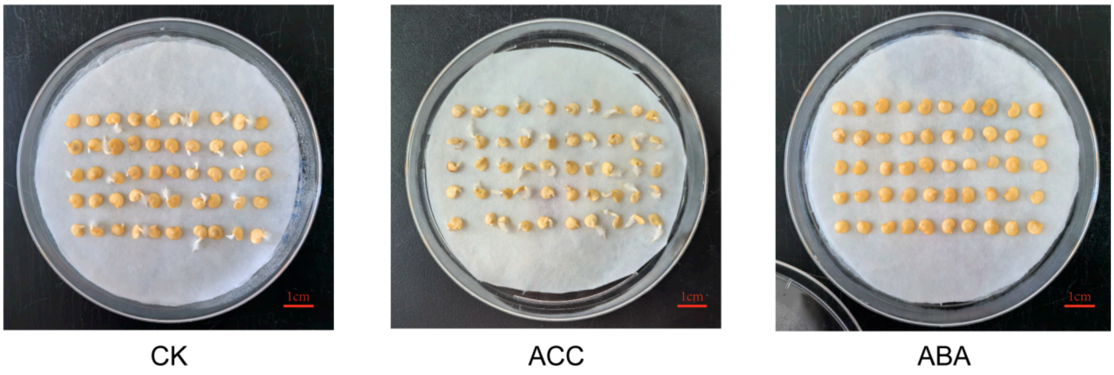

Figure S1. Germination of pepper seeds under dH_2O (CK), ACC and ABA treatments. (a) Germination of pepper seeds, with the x-axis representing treatment time and the y-axis representing seed germination. All experiments were conducted in triplicate with at least three independent biological replicates. Error bars indicate the standard error of the mean. A t-test was used to determine statistically significant differences in the germination under different conditions compared to CK (* $P < 0.05$, ** $P < 0.01$). (b) Representative images of seed germination morphology after 72 hours of treatment.
